# Design and Application of Microfluidic Capture Device for Physical–Magnetic Isolation of MCF-7 Circulating Tumor Cells

**DOI:** 10.3390/bios14060308

**Published:** 2024-06-15

**Authors:** Akhilesh Bendre, Derangula Somasekhara, Varalakshmi K. Nadumane, Ganesan Sriram, Ramesh S. Bilimagga, Mahaveer D. Kurkuri

**Affiliations:** 1 Centre for Research in Functional Materials, JAIN (Deemed-to-be University), Bengaluru 562112, Karnataka, India; akhilesh.bendre@jainuniversity.ac.in; 2 Department of Biotechnology, School of Sciences, JAIN (Deemed-to-be-University), JC Road, 34, 1st Cross Road, Sudharna Nagar, Bengaluru 560027, Karnataka, India; d.somasekhara@jainuniversity.ac.in (D.S.); kn.varalakshmi@jainuniversity.ac.in (V.K.N.); 3 School of Chemical Engineering, Yeungnam University, Gyeongsan 38541, Republic of Korea; sriramyu@yu.ac.kr; 4 Department of Minimal Access, GI and Bariatric Surgery, Fortis Hospital, 14, Cunningham Road, Bengaluru 560052, Karnataka, India; bilimaga@hcgel.com; 5 Interdisciplinary Research Centre (IDRC), JAIN (Deemed-to-be University), Jain Global Campus, Bengaluru 562112, Karnataka, India

**Keywords:** microfluidics, CTCs, capture, physical-bio affinity based/guided magnetic isolation, functionalized nanoparticles, simulation-aided

## Abstract

Circulating tumor cells (CTCs) are a type of cancer cell that spreads from the main tumor to the bloodstream, and they are often the most important among the various entities that can be isolated from the blood. For the diagnosis of cancer, conventional biopsies are often invasive and unreliable, whereas a liquid biopsy, which isolates the affected item from blood or lymph fluid, is a less invasive and effective diagnostic technique. Microfluidic technologies offer a suitable channel for conducting liquid biopsies, and this technology is utilized to extract CTCs in a microfluidic chip by physical and bio-affinity-based techniques. This effort uses functionalized magnetic nanoparticles (MNPs) in a unique microfluidic chip to collect CTCs using a hybrid (physical and bio-affinity-based/guided magnetic) capturing approach with a high capture rate. Accordingly, folic acid-functionalized Fe_3_O_4_ nanoparticles have been used to capture MCF-7 (breast cancer) CTCs with capture efficiencies reaching up to 95% at a 10 µL/min flow rate. Moreover, studies have been conducted to support this claim, including simulation and biomimetic investigations.

## 1. Introduction

Cancer is one of the major diseases responsible for around 10 million annual deaths worldwide in the year 2022 [1]. The diagnosis of cancer is one of the more complex and expensive aspects of medical care [2]. The conventional methods employed for cancer diagnosis usually involve the use of an invasive biopsy technique directly from the affected tissue followed by various visual and immunological tests. In certain cases where the aforementioned test is not possible, a blood biopsy is performed involving the collection of large blood sample followed by separation techniques, after which immunological as well as visual confirmations are performed. These methods are very time-consuming and labor-intensive [3,4]. A liquid biopsy offers a less invasive method of collecting affected biological entities, often directly from a blood sample. Among the various cellular entities that can be isolated, circulating tumor cells (CTCs) are one of the most important entities. CTCs and their population can indicate the nature of cancer and the degree of its invasion in the body. The isolation of CTCs from blood offers much more efficient and less invasive ways to conduct liquid biopsies. As the utilization of immunological or physical capture without the use of a modulated microenvironment or a micro device (i.e., at the macro-scale) is very difficult and unreliable, microfluidic technology provides an ideal solution for such problems [5,6].

Microfluidic technology has been a field of great interest over the past two decades, and it has been used in various technologies such as in the rapid synthesis of nanoparticles, efficient separation, the enrichment of various cellular entities for biomedical analysis and as a highly adjustable platform for bio-sensing and bio-mimicking studies [7]. Microfluidic techniques are specifically targeted towards the manipulation of extremely small quantities of fluid and microscale entities with a very high degree of precision, and they provide a very flexible platform for various applications on a single chip. The manipulation of small-scale entities is very difficult to achieve using microscale tools (such as micro-tweezers or needles) in a macroenvironment (such as Petri dishes). Microfluidic chips have been utilized to capture biological entities, such as CTCs, exosomes and deoxyribonucleic acid (DNA)/ribonucleic acid (RNA), for a few decades [8,9,10,11]. Various techniques have been used to capture CTCs, but these can be divided into two major classifications, namely (i) physical and (ii) immunological capture. The physical methods include using the physical properties (such as their larger size, larger mass and different shape) of CTCs to isolate them [12]. These techniques include hydrodynamic flow focusing, entrapment using micro/nanostructures, dielectrophoresis (DEP) and acoustic isolation [13,14,15]. Immunological/bio-affinity methods involve utilizing the CTC membrane’s entities, such as molecules (such as the epithelial cell adhesion molecule (EpCAM)), proteins, receptors and antigens, to chemically interact with functionalized nanoparticles and chip surfaces to trap them [11]. Immunomagnetic/bio-affinity capture methods can be placed in such a category. These methods generally utilize magnetic nanoparticles (MNPs) that are functionalized with antibodies and other chemically relevant molecules, such as folic acid, to produce functionalized nanoparticles, which can chemically bind to the CTC’s membrane. Based on the type of magnetic material (ferromagnetic, diamagnetic and paramagnetic) used and the magnetic field strength used, the now MNP-covered CTCs will move in a specific way (based on attractive or repulsive interaction), which will eventually isolate the CTCs from other types of cells [16,17,18]. In recent years, various techniques have been proposed to isolate CTCs. Cha et al. proposed a novel method for continuous CTC isolation using lateral magnetophoresis and magnetic nanoparticles functionalized with both EpCAM and vimentin antibodies along with a mesenchymal marker. The results indicate that using both antibodies significantly improved CTC isolation compared to using either of the biomarkers alone [19]. Jiang et al. constructed hybrid engineered cell membrane-camouflaged magnetic nanoparticles (HE-CM-MNs) obtained from a Jurkat cell and lentivirus for the highly efficient capture of heterogeneous CTCs with high purity, which was enabled by inheriting the recognition ability of HE-CM for various CTCs and reducing homologous cell interaction with leukocytes [20]. It is known that cancerous cells, especially MCF-7 breast cancer cells, have a very high concentration of folate receptors on their cell membrane (up to 100 to 300 times compared to normal cells), which can be used as very sensitive biomarkers for cancerous cells. This overexpression can be utilized to attach a large amount of modified nanoparticles to obtain the desired effect, which will have a minimum impact on normal blood or tissue cells [21,22]. Ren et al. used folic acid- and hyaluronic acid-modified carbon dot-coated magnetic nanoparticles (CD@NMs) with the overexpressed folate receptor on tumor cells and the self-propulsion of CD@NMs under the drive of a magnetic field and H_2_O_2_, CD@NMs can efficiently and sensitively capture CTCs [23]. Bio-affinity capture methods are very effective isolation methods but can be combined with physical methods to increase capture efficiency. The primary considerations to perform efficient entrapment are the chip design (which is easy to use, and the isolated CTCs should be easily recoverable), the functionalization of nanoparticles and testing the working principle [16,24,25]. Before conducting an experimental study using a microfluidic chip, the physics simulation software can be used to predict the nature of fluid flow and the behavior of the particles. These software can predict various effects and parameters of the experiment with a high degree of certainty [26].

This work uses a novel microfluidic chip and functionalized MNPs to isolate MCF-7 breast cancer cells in a simulated blood environment, mimicking CTCs in a blood sample. Fe_3_O_4_ nanoparticles have been functionalized with folic acid to use the overexpression of folate receptors in MCF-7 breast cancer cells to capture them. This study aims to design and exhibit the function of the chip to capture CTCs with high efficiency. The chip design used in this study and the method for functionalizing Fe_3_O_4_ nanoparticles have not yet been reported to the best of the authors’ knowledge.

## 2. Materials and Methods

### 2.1. Materials

Ferric chloride (FeCl_3_) (97%) and sodium hydroxide (NaOH) pellets (98%) were purchased from AVRA (Science World chemical suppliers, Bengaluru, Karnataka, India) Pvt. Ltd. N-hydroxysuccinimide (NHS) (99%), 1,2-dichloroethane (ethylene dichloride [EDC]) (99%), ferrous chloride (FeCl_2_) (98%), Dulbecco’s Modified Eagle Medium (DMEM), dopamine hydrochloride (98%), folic acid (FA) (97%), micro particles based on polystyrene (10 and 20 µm) and Tris-HCl buffer were purchased from Sigma Aldrich (Science World chemical suppliers, Bengaluru, Karnataka, India) Pvt. Ltd. Polydimethylsiloxane (PDMS) (SYLGARD 184 silicone elastomer and curing agent) was used for device fabrication, and it was purchased from Dow Corning (Science World chemical suppliers, Bengaluru, Karnataka, India) Pvt. Ltd. MCF-7 breast cancer cells were acquired from the Department of Biotechnology, JAIN (Deemed-to-be-University), Bengaluru, Karnataka, India.

### 2.2. Functional Nanoparticle Synthesis

#### 2.2.1. Magnetic Nanoparticle (MNP) Synthesis

Fe_3_O_4_ nanoparticles (MNPs) were obtained via a simple co-precipitation method involving the mixing of 1.71 g of ferrous chloride (FeCl_2_) and 2.32 g of ferric chloride (FeCl_3_) salts in 80 mL of water. The mixture underwent vigorous stirring at 85 °C for 30 min. Then, 2 M of sodium hydroxide (NaOH) solution was added to the mixture drop-wise until a pH level of 10 was achieved. Then, the mixture was stirred at 85 °C for 30 min. The mixture was cooled to room temperature, and the precipitate was separated and washed using the magnetic and centrifugation methods. Furthermore, it was dried overnight in a vacuum oven at 60 °C, and the dried precipitate was crushed into a fine powder to obtain the magnetic nanoparticles (MNPs) [27]. The obtained MNPs were labeled as FO.

#### 2.2.2. Functionalization of MNP

The functionalization of Fe_3_O_4_ nanoparticles was carried out in a two-step process. For polydopamine coating (PDA), 300 mg of Fe_3_O_4_ nanoparticles was suspended in 60 mL of dopamine hydrochloride solution (1 mg/mL) in 10 mM Tris-HCl buffer (pH 8.0) under vigorous stirring for 4 h at room temperature. The obtained precipitate was washed and dried overnight. It was labeled as FO-PDA. Folic acid was functionalized over the PDA-coated Fe_3_O_4_ nanoparticles using EDC-NHS coupling. PDA-coated Fe_3_O_4_ nanoparticles (250 mg) and FA (200 mg) were mixed in 20 mL of 10 mM Tris-HCl buffer (pH 8.0). NHS (50 mg) and EDC (100 mg) were added to the mixture and stirred for 4 h at room temperature under a dark environment. The precipitate was magnetically separated, washed, dried and stored in 0.2 M phosphate-buffered solution (PBS) (pH: 7.2). It was labeled as FO-PDA-FA [28].

### 2.3. Microfluidic Chip and Experimental Setup

**Figure 1:**
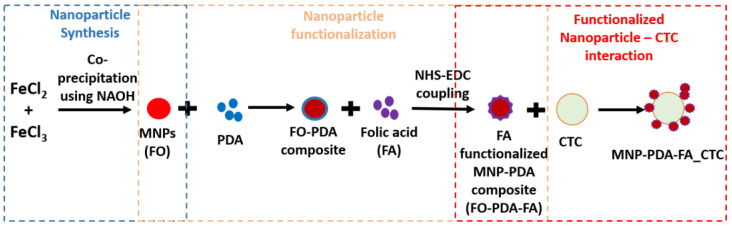
The synthesis route utilized for the preparation of folic acid-functionalized Fe_3_O_4_ nanoparticles via chemical synthesis followed by organic functionalization and their consequent interaction with folate receptors on the CTCs’ membranes.

**Figure 2:**
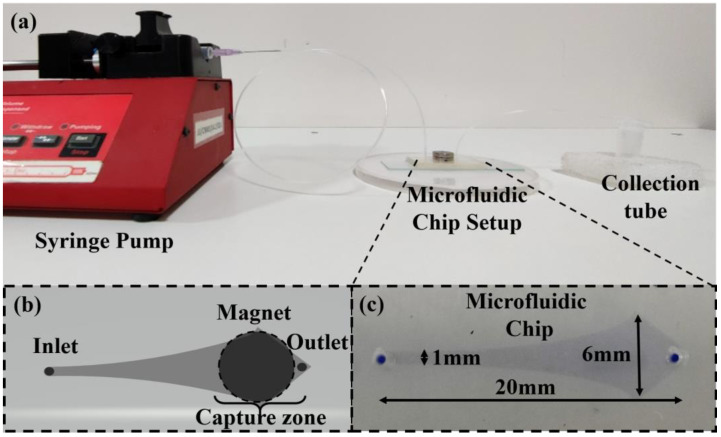
The capture technique setup of (**a**) the actual experimental setup for the capture of CTCs. (**b**) A schematic diagram of the microfluidic chip setup. (**c**) The actual microfluidic channel (the channel is dyed with methylene blue solution to provide ease of visibility).

The microfluidic chip was prepared in the AutoCAD 2016 software, and the pattern was fabricated using the standard photolithographic technique with a channel consisting of a narrow pattern that gradually expands in cross-sectional area, plasma-bonded to a glass slide (as shown in Figure 1). The microchannel depth is 100 µm, whereas the width from the inlet to the outlet varies from 1 mm (Min.) to 6 mm (Max.), respectively, and the total length from the inlet to the outlet is 20 mm. The setup consists of an inlet connected via a polytetrafluoroethylene (PTFE) tube (outer diameter: 1 mm) to a standard 2 mL syringe mounted on a syringe pump (as shown in Figure 2a,c). A cylindrical magnet is placed on the chip and can be removed or placed physically (as shown in Figure 2b).

### 2.4. Characterization

All of the materials (FO, FO-PDA and FO-PDA-FA) were analyzed with various analytical techniques to understand their physical and chemical properties. The surface morphology was determined by utilizing the field emission scanning electron microscopic (FESEM) technique (HITACHI SU-70, Japan). For the study of structural-crystalline features, powder X-ray diffraction (XRD) patterns for the samples were recorded on an Ultima-IV X-ray diffractometer (M/s. Rigaku Corporation, Japan) with Ni-filtered Cu Kα radiation (1 = 1.5406 Ao) at a 2θ scan speed of 2 degrees/min and a scan range of 5 to 80 degrees at 40 KV and 30 Ma. X-ray photoelectron spectroscopy (XPS) was used to analyze the chemical states of the elements in the sample using VG multi-lab 2000, which was operated at 3.1 meV using an Al-Kα as the energy source. Olympus CKX41 (inverted microscope) was used with a regular 10 MP phone video camera to record pictures and videos of the capture experiments.

### 2.5. Operating Principle: Simulation, Biomimetic Study and Capture Study

The capture of CTCs is based on (a) the physical technique established based on the particle–fluid interaction and (b) the bio-affinity-based magnetic technique based on the functionalized nanoparticle interaction with CTCs. Multiple forces are acting on a particle in a microfluidic environment, such as forces that promote the flow of the particle in the fluid (local acceleration due to fluid pressure and velocity) and opposing forces such as the drag force, lift forces (due to the fluid and walls of the chip) and forces due to particle–particle interaction (collisions and particle surface interactions due to electrostatic interactions). The culmination of these forces acting on particles with different physical attributes leads to different flow patterns and behaviors, further based on the flow rate and chip design. Physical characteristics, such as the size, mass, and surface morphology, may contribute to physical capture. In contrast, chemical attributes such as the overexpression of cell membrane surface moieties can lead to bio-affinity-based capture. The presence of many receptors, such as folate receptors on the surface, allows for molecules such as folic acid to interact with and bind to the receptor. This can be utilized to capture CTCs directly onto the chip. Still, these molecules can also be functionalized onto nanoparticles with electric and magnetic properties, which can then be manipulated to capture CTCs. This work utilizes a similar method where MNPs are functionalized with folic acid and will interact with a large number of folate receptors that are present on the CTCs’ membranes.

Simulation studies are performed using the COMSOL Multiphysics v 5.5 software to test the hypothesis of physical capture. The design was developed in a CAD kernel format and tested using a fluid flow module (specifically creeping flow and particle tracing for fluid flow). The particle size of 20 µm and forces such as the Stokes drag and wall-induced lift forces were considered. The design material was chosen to be water at room temperature, and a flow rate equivalent to the lowest flow rate (10 µL/min) was considered. Two studies were computed, namely stationary and time-dependent studies, to obtain the fluid velocity–pressure profile and particle trajectories, respectively.

The biomimetic study was performed using polystyrene beads of two different hydrodynamic sizes (20 µm and 10 µm to mimic the average physical sizes of MCF-7 cells (CTCs) and WBCs-RBCs, respectively). The concentration of the 20 µm sized beads was lower than that of the 10 µm sized beads (a ratio of 1:100 for 20 µm to 10 µm beads was prepared and further diluted to be used). This study was performed to check whether the results of the simulation study match the real-time flow profile of the beads and to verify the physical trapping potential of the chip design. The primary considerations were the individual density of the beads and the lack of any potential surface moieties, which may cause the beads to group into a bigger entity. It can be logically assumed that the density of CTCs is much higher than that of polystyrene, and CTCs may be prone to the formation of large clumps.

CTC capture studies were performed using MCF-7 breast cancer epithelial cells as these have a very narrow and consistent size range, with the average size being 20 µm, as well as the overexpression of folate receptors on their cell membrane, which is ideal for immunological binding with MNPs functionalized with folic acid [29]. The studies were performed by counting the number of CTCs in a batch and then dividing them into 22 batches of 250 µL each with equal concentrations of CTCs. One batch was kept as the control, and the others were treated as experimental batches, i.e., 16 for flow rate studies and 5 for nanoparticle/sample ratio studies. Each batch contained an average of 125,000 cells (counted using a hemocytometer). All of the suspensions were synthesized using Dulbecco’s Modified Eagle Medium (DMEM). The flow rate studies were performed by flowing each batch at different flow rates, such as 10, 20, 30, 40, 50, 60, 70 and 80 µL/min, and then counting the cells obtained in the collection tube (denoted by x). The studies were performed in the absence and presence of a magnetic field. The functionalized nanoparticles were centrifuged, dried, weighed and then suspended in the media (DMEM) to achieve a concentration of 100 ppm (1 mg/mL). The obtained suspension was mixed in appropriate ratios of 1:1, 1:2, 1:3, 1:4 and 1:5 of nanoparticle suspension volume to sample volume. The experiment was performed similarly to the flow rate experiment at a constant flow rate of 40 µL/min. The capture percentage was calculated as mentioned before.

## 3. Results

### 3.1. Characterization

**Figure 3:**
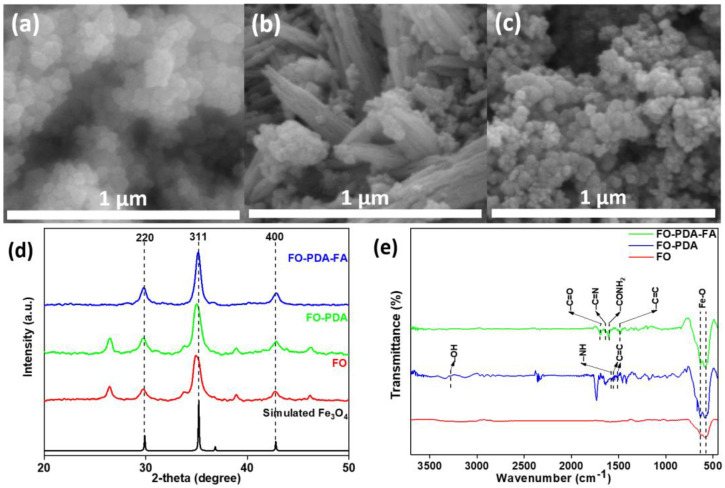
The FESEM images of (**a**) Fe_3_O_4_ nanoparticles (FO), (**b**) Fe_3_O_4_ particles embedded in PDA (FO-PDA) and (**c**) Fe_3_O_4_-PDA nanoparticles functionalized with folic acid (FO-PDA-FA). (**d**) XRD spectra and (**e**) FTIR spectra of FO, FO-PDA and FO-PDA-FA.

The FESEM analysis visually confirmed the formation of spherical Fe_3_O_4_ nanoparticles with particle sizes of approximately 80 nm, as seen in Figure 3a. In contrast, in Figure 3b, PDA fibers along with the particles can be seen (due to an excess of PDA utilized to ensure the complete synthesis of the FO-PDA sample). Figure 3c shows clusters of spherical particles with sizes similar to the ones shown in Figure 3a, but later, the FT-IR data confirm the presence of groups which cannot be assigned to FO nor FO-PDA samples, but they are expected to be derived from folic acid, thus confirming its presence. The XRD analysis for samples of Fe_3_O_4_ nanoparticles coated in PDA and folic acid, shown in Figure 3d, have characteristic peaks for Fe_3_O_4_ corresponding to the crystal planes of (2 2 0), (3 1 1) and (4 4 0), which reveal that even after surface modification, the nanoparticles were Fe_3_O_4_ with a cubic structure [30,31]. The FTIR spectrum obtained from FO (MNPs), FO-PDA and FO-PDA-FA is presented in Figure 3e, confirming the surface modification of the MNPs. The two noticeable peaks seen at 585 cm^−1^ and 633 cm^−1^ for all samples can be attributed to the stretching vibration mode of the metal–oxygen (Fe–O) bonds present in the crystalline framework of Fe_3_O_4_. These peaks are typical features of spinel structures, namely ferrites. In the case of FO-PDA, the peak at 3232 cm^−1^ is associated with the stretching vibration of O–H, while the peak at 1584 cm^−1^ is identified as the stretching as well as bending vibration of the N–H group and the characteristic peaks at 1561 and 1509 cm^−1^ are linked to the –C=C– bonds coining from the indole structure of PDA. For the FO-PDA-FA sample, the peak at 1694 cm^−1^ can be observed for the (–C=O) group, which is related to the pterin structure. The peaks at around 1635 cm^−1^ and 1597 cm^−1^ belong to the stretching (–C=N) and bending of (–CONH2), respectively. The peak at 1477 cm^−1^ pertains to the (–C=C) stretching of phenyl and pterin rings [31,32,33].

**Figure 4:**
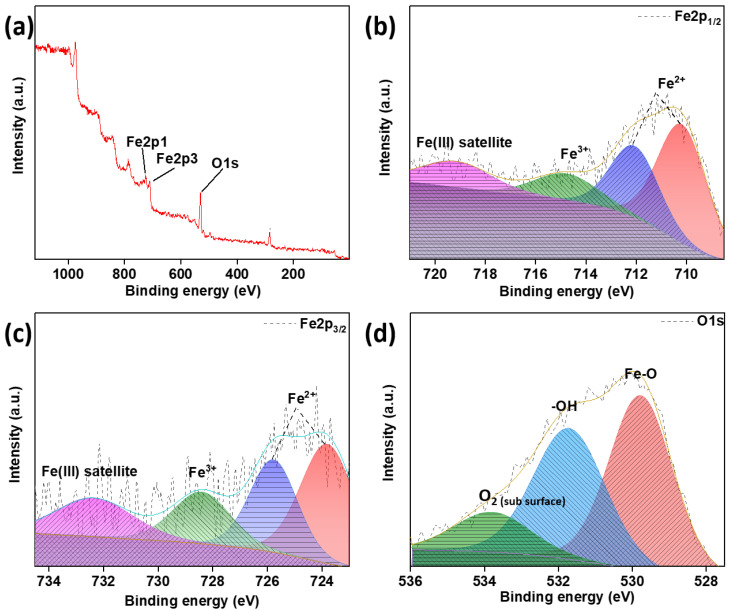
XPS spectra of Fe_3_O_4_ nanoparticles (FO). (**a**) Survey spectra, high-resolution spectra of (**b**) Fe2p_1/2_, (**c**) Fe2p_3/2_ and (**d**) O1s.

Furthermore, an XPS analysis was performed to confirm the formation of MNPs, as shown in Figure 4. As can be seen from the survey spectra, the elements presented are oxygen and ferrous and ferric ions with peaks present at 529 eV, 725 eV and 710 eV, respectively. The same can be further confirmed by the high-resolution spectra of Fe^2p^, confirming the presence of two bonds for Fe^2+^ and a single bond for Fe^3+^. The high-resolution spectra of O1s also show the presence of Fe–O and –O–H bonds and subsurface O_2_ at 529.7, 531.6 and 534 eV, respectively [30]. The Fe-O bond as well as the presence of both oxidation states of iron (Fe) confirms the presence of Fe_3_O_4_ nanoparticles. The –OH bond, as well as the sub surface O_2_, shows the presence of adsorbed water and O_2_ adsorbed at tetrahedral or octahedral sites in the crystal.

### 3.2. Simulation and Biomimetic Studies

**Figure 5:**
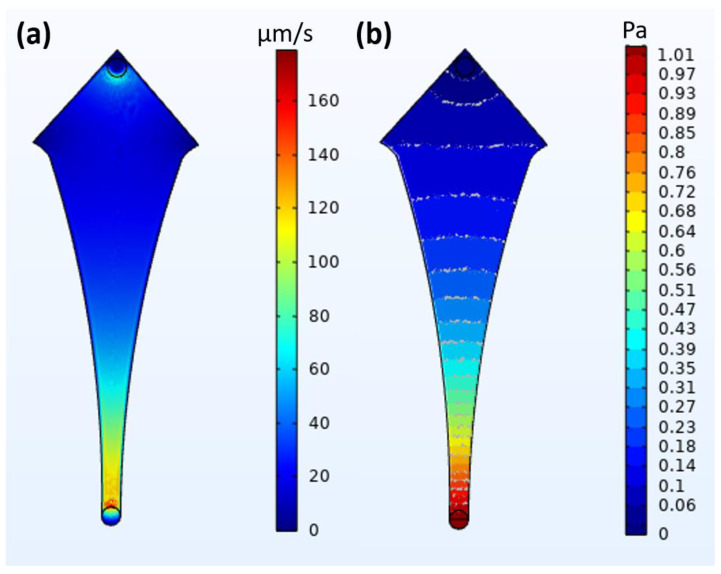
Fluid dynamics-based simulation of aqueous fluids to determine (**a**) velocity of flow (flow rate) and (**b**) pressure profile of fluid.

The simulation studies performed for the fluid velocity and pressure profile are shown in Figure 5. As can be seen, the fluid velocity is the highest at the inlet (the junction of the inlet tube and microchannel, shown as dark red in Figure 5a). The velocity then reduces rapidly as it approaches the device’s most expansive area, almost reaching zero at the extreme corners of the capture zone (shown as dark blue in Figure 5a). The pressure profile shows a similar phenomenon: the pressure is the greatest (going above 1 Pa) at the inlet (shown as dark red in Figure 5b) but reduces rapidly and becomes zero at the widest region (shown as dark blue in Figure 5b). These profiles help us understand and predict how the fluid flow affects the particles in this specific design, as fluid velocity and pressure are the major driving forces of particle movement.

**Figure 6:**
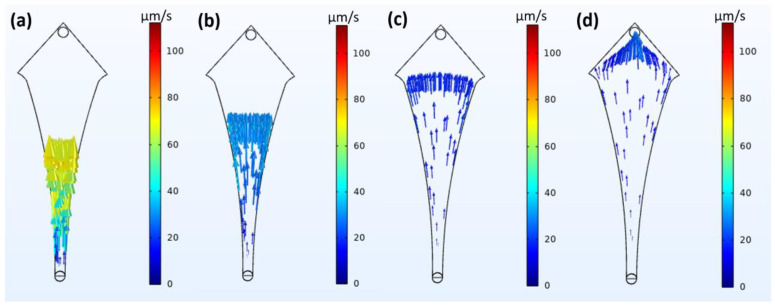
Fluid–particle interaction-based simulation of particles in flow to determine particle trajectories at different times given as (**a**) 60 s, (**b**) 120 s, (**c**) 360 s and (**d**) 480 s.

The simulation studies performed for particle trajectories at various times are shown in Figure 6 and Video S1. As shown, the particles follow the trajectories expected from the velocity and pressure profiles but retain their velocity for a longer duration after release. The particles move very rapidly as they enter from the inlet (shown as yellow in Figure 6a) but slow down rapidly as they approach the widest region (shown as dark blue in Figure 6b,c), reducing their velocities to almost zero. It can also be seen that most particles shift into the corner regions and tend to stay in the regions for a relatively long time as there are very weak forward driving forces, with the fluid velocity and pressure almost being zero (shown in Figure 6d). Biomimetic studies were performed and recorded, and the results are presented in Video S2. It can be seen that the 20 µm sized beads tend to slow down by a very large degree compared to their 10 µm counterparts and move towards the corner regions, where a reasonable amount of them eventually stop.

### 3.3. CTC Capture Study

**Figure 7:**
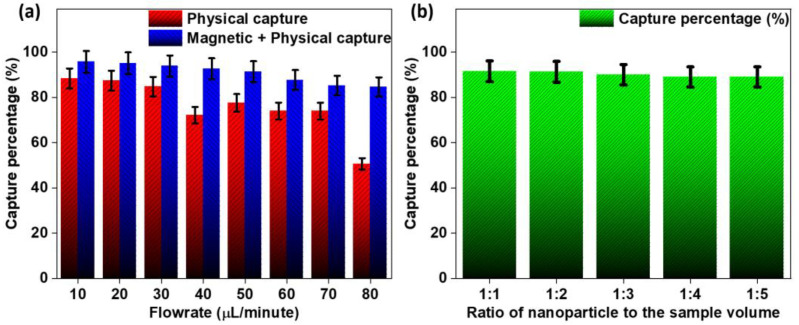
Capture percentages were obtained for (**a**) only physical as well as physical–immunomagnetic capture at different flow rates and (**b**) different ratios of nanoparticle to sample volume.

CTC capture studies were performed, and the obtained results are presented in Figure S1, Video S3 and Figure 7. Figure S1 and Video ESM3 show the capture of CTCs under the absence and presence of a magnetic field (Video S3b–d), respectively. As can be seen, the physical capture of CTCs is possible with a high degree of capture, but the use of bio-affinity-based magnetic capture methods can enhance the capture rate. As shown in Figure 7b, the nanoparticle-to-sample volume ratio does not vary significantly from 1:1 to 1:5 as it decreases very gradually, with a major drop at a 1:3 ratio. The capture studies performed in the absence and presence of the magnetic field show that physical capture is viable up to a rate of 40 µL/min (above 90% capture), after which it decreases rapidly, reaching almost 50% at 80 µL/min. The magnetic–physical capture shows the highest capture rate at 10 µL/min, but unlike the physical-only capture, it decreases gradually with an increase in the flow rate. The capture rate stays viable up to 50 µL/min, staying above a 90% capture efficiency.

## 4. Discussion

The various characterization techniques confirm the synthesis and surface modification of Fe_3_O_4_ nanoparticles. The FESEM analysis shows the nanoparticles in an almost spherical structure. The absence of fibers in the FO-PDA-FA samples may be due to the removal of excess PDA before and after functionalization with folic acid (FA). The XRD analysis shows that Fe_3_O_4_ nanoparticles are present even after modifications. The results show that the FTIR analysis confirmed that the surface modification was performed correctly. The PDA, as well as the folic acid, was successfully functionalized layer by layer over the Fe_3_O_4_ nanoparticles. The XPS analysis further confirms the formation and presence of Fe_3_O_4_ nanoparticles.

The simulation studies predict with high certainty the nature and trajectories of the fluid flow and the particles, respectively. The fluid velocity and pressure drive the particles towards the outlet. Still, simultaneously, the drag force on the particle resists its movement, and the lift forces prevent them from flowing in a straight line, pushing them towards the corner regions. The pressure regions (wavefront) also further pushed the particles towards the corners, into areas of minimal/inactive flow. The time-dependent particle trajectory study indicates that most particles behave as predicted by stationary studies. This theory is further solidified by biomimetic research, where it can be seen that the 20 µm particles slow down by a large factor and even stop in the corner regions compared to the 10 µm particles. These aforementioned effects can be translated to CTCs, but the effects shown here may be more effective due to the physical properties of the CTCs, such as relative density, adhesion to surfaces, etc. The sudden drop in the flow rate, as well as the velocities of the particles along with the specific design of the chip, may create regions of inactive flow, which are more prominent towards the corner regions. This may be considered as a means of physically capturing tumor cells (without the use of traps), in the least acting as a physical enhancement to bio-affinity-based magnetic capture.

The capture studies indicate that bio-affinity-based enhancement improves the overall capture efficiency by up to an average of 10–12% compared to physical-only capture. The enhancement is also viable for higher flow rates (>40 µL/min). In contrast, physical capture fails because the fluid velocity in the capture region is high enough to push the CTCs towards the outlet. Bio-affinity-based enhancement combined with the physical capture strategy can produce a capture rate of above 90%. The amount of nanoparticles added to the CTC sample can be as low as one-third of the whole sample volume to produce an optimal capture rate. In real-time use, it might be possible to reduce this even further as the concentration of CTCs in a whole blood sample is usually significantly less.

## 5. Conclusions

The novel microfluidic chip and method of capture shown in this work have been successfully employed towards capturing MCF-7 CTCs with the highest efficiency of 95.6%. The combined physical and bio-affinity-based magnetic capture methods synergized to boost the capture efficiency of a simple-to-use chip. Simulation and biomimetic studies were performed to understand and prove that the physical capture hypothesis aligns with the experiments performed with CTCs. Microfluidic technology offers various practical advantages over conventional biopsies, with the different methods of capturing CTCs only being limited by the complexity of chip designs; with huge leaps in material, biology sciences, the development of ultra-sensitive machinery and the advent of autonomous processes, this technology can be further improved towards single-cell capture on an everyday commercial scale.

## Data Availability

The data that support the findings of this study are available within this article [and its Supplementary Materials].

## Supplementary Materials

The following supporting information can be downloaded at: https://www.mdpi.com/article/10.3390/bios14060308/s1, Figure S1. An enlarged view of CTC capture observed at 10 µL/min for (a) the region just after the inlet, (b,c) the corner regions in the capture area without a magnetic field and (d) the corner region in the capture area under a magnetic field (please check associated Video S3 (a–d)); Video S1. An animated simulation of particle trajectories in the chip at 10 µL per min; Video S2. An enlarged view of polystyrene bead flow in the chip at 10 µL per min; Video S3. (a) An enlarged view of CTC capture observed at 10 µL per min for the region just after the inlet; Video S3. (b) An enlarged view of CTC capture observed at 10 µL per min for the corner region in the capture area without a magnetic field; Video S3. (c) An enlarged view of CTC capture observed at 10 µL per min for the corner region in the capture area without a magnetic field; Video S3. (d) An enlarged view of CTC capture observed at 10 µL per min for the corner region in the capture area under a magnetic field.
