# Supplementary material for: Design and Application of Microfluidic Capture Device for Physical–Magnetic Isolation of MCF-7 Circulating Tumor Cells
Source: Biosensors (Basel). 2024 Jun 15;14(6):308. doi: 10.3390/bios14060308 (PMC11201624; doi:10.3390/bios14060308)
Supplement: Supplementary file 1 [file biosensors-14-00308-s001.zip › biosensors-3025732-supplementary.pdf]

biosensors

Supplementary Materials

Design and application of microfluidic capture device for physical-immunomagnetic isolation of MCF-7 circulating tumour cells

Akhilesh Bendre[1], Derangula Somasekhara[2], Varalakshmi K.Nadumane[2], Ganesan Sriram[3], Ramesh S. Bilimagga[4], Mahaveer D. Kurkuri[1, 5, *]

[1] Centre for Research in Functional Materials, JAIN (Deemed-to-be University), Bengaluru, Karnataka-562112, India; akhilesh.bendre@jainuniversity.ac.in
[2] Department of Biotechnology, JAIN (Deemed-to-be-University), School of Sciences, JC Road, 34, 1st Cross Road, Sudharna Nagar, Bengaluru, Karnataka- 560027, India; kn.varalakshmi@jainuniversity.ac.in, d.somasekhara@jainuniversity.ac.in
[3] School of Chemical Engineering, Yeungnam University, Gyeongsan 38541, Republic of Korea; sriramyu@yu.ac.kr
[4] Department of Minimal Access, GI and Bariatric Surgery, Fortis Hospital, 14, Cunningham Road, Bengaluru, Karnataka -560052, India; bilimaga@gmail.com
[5] Interdisciplinary Research Centre (IDRC), JAIN (Deemed-to-be University), Jain Global Campus, Bengaluru, 562112, Karnataka, India; mahaveer.kurkuri@jainuniversity.ac.in
[*] Correspondence: mahaveer.kurkuri@jainuniversity.ac.in

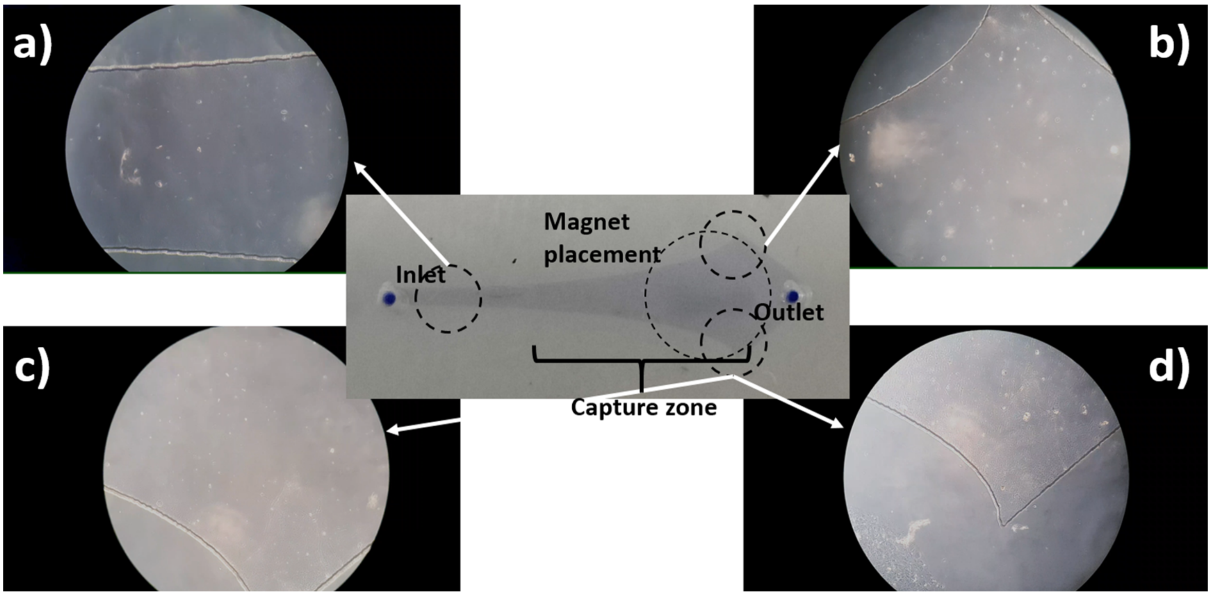

Figure S1. Enlarged view of CTC capture observed at 10 µl/min for a) region just after the inlet, b), c) the corner regions in the capture area without magnetic field and d) the corner region in the capture area under a magnetic field. (Please check associated **Video S3 a), b), c) and d)**).
